# Long-Term Variations of Global Solar Radiation and Its Potential Effects at Dome C (Antarctica)

**DOI:** 10.3390/ijerph19053084

**Published:** 2022-03-06

**Authors:** Jianhui Bai, Xuemei Zong, Christian Lanconelli, Angelo Lupi, Amelie Driemel, Vito Vitale, Kaili Li, Tao Song

**Affiliations:** 1 LAGEO, Institute of Atmospheric Physics, Chinese Academy of Sciences, Beijing 100029, China; zxm@mail.iap.ac.cn; 2 European Commission, Joint Research Centre, Via Fermi 2749, 21027 Ispra, Italy; christian.lanconelli@ec.europa.eu; 3 Institute of Polar Sciences (CNR-ISP), National Research Council of Italy, Via P. Gobetti 101, 40129 Bologna, Italy; a.lupi@isac.cnr.it (A.L.); v.vitale@isac.cnr.it (V.V.); 4 Alfred Wegener Institute, Helmholtz Centre for Polar and Marine Research, Am Handelshafen, 12, 27570 Bremerhaven, Germany; amelie.driemel@awi.de; 5 Nanjing Zhongkehuaxing Emergency Science and Technology Research Institute, Nanjing 211899, China; 20171201073@nuist.edu.cn (K.L.); songtao_1985@tom.com (T.S.)

**Keywords:** absorbing and scattering substances, energy balance, air temperature, albedo, climate and climate change

## Abstract

An empirical model to predict hourly global solar irradiance under all-sky conditions as a function of absorbing and scattering factors has been applied at the Dome C station in the Antarctic, using measured solar radiation and meteorological variables. The calculated hourly global solar irradiance agrees well with measurements at the ground in 2008–2011 (the model development period) and at the top of the atmosphere (TOA). This model is applied to compute global solar irradiance at the ground and its extinction in the atmosphere caused by absorbing and scattering substances during the 2006–2016 period. A sensitivity study shows that the responses of global solar irradiance to changes in water vapor and scattering factors (expressed by water vapor pressure and S/G, respectively; S and G are diffuse and global solar irradiance, respectively) are nonlinear and negative, and that global solar irradiance is more sensitive to changes in scattering than to changes in water vapor. Applying this empirical model, the albedos at the TOA and the surface in 2006–2016 are estimated and found to agree with the satellite-based retrievals. During 2006–2016, the annual mean observed and estimated global solar exposures decreased by 0.05% and 0.09%, respectively, and the diffuse exposure increased by 0.68% per year, associated with the yearly increase of the S/G ratio by 0.57% and the water vapor pressure by 1.46%. The annual mean air temperature increased by about 1.80 °C over the ten years, and agrees with the warming trends for all of Antarctica. The annual averages were 316.49 Wm^−2^ for the calculated global solar radiation, 0.332 for S/G, −46.23 °C for the air temperature and 0.10 hPa for the water vapor pressure. The annual mean losses of solar exposure due to absorbing and scattering substances and the total loss were 4.02, 0.19 and 4.21 MJ m^−2^, respectively. The annual mean absorbing loss was much larger than the scattering loss; their contributions to the total loss were 95.49% and 4.51%, respectively, indicating that absorbing substances are dominant and play essential roles. The annual absorbing, scattering and total losses increased by 0.01%, 0.39% and 0.28% per year, respectively. The estimated and satellite-retrieved annual albedos increased at the surface. The mechanisms of air-temperature change at two pole sites, as well as a mid-latitude site, are discussed.

## 1. Introduction

The Intergovernmental Panel on Climate Change (IPCC) reports mean global warming as 0.6 ± 0.2 °C during the 20th century, and anthropogenic increases in greenhouse gases are the likely cause of this temperature rise over the last 50 years [1]. The annual mean temperatures on the Antarctic Peninsula have risen rapidly since recordkeeping began in the 1950s [1,2,3]. The total increase in the annual mean air temperature of 2.8 °C makes it the most rapidly warming region in the southern hemisphere, comparable to rapidly warming regions of the Arctic [4]. For the 19 stations in the Antarctic Peninsula over 1951–2000, 11 had warming trends and 7 had cooling trends in their annual surface temperature [2]. Several possible mechanisms are discussed, e.g., changing oceanographic or atmospheric circulations, or a regional air-sea-ice feedback mechanism amplifying the greenhouse warming effects. However, we still lack a sound basis for predicting climate change in this region [1]. A more recent study reports that there was a shift from a warming trend of 0.32 °C/decade during 1979–1997 to a cooling trend of −0.47 °C/decade during 1999–2014 [5]. In short, mechanisms driving and modulating warming in Antarctica are not clear. The mean values of surface air temperature and other meteorological variables are the result of many processes and interactions involving physical and chemical atmospheric features that took place over several decades [3]. Therefore, long-term site-process studies related to the climate and climate change are still necessary. Solar radiation is the fundamental energy source for the earth–atmosphere system. Thus, its transfer and distribution in the atmosphere, and its reflection at the surface and the top of the atmosphere (TOA), should be investigated thoroughly.

Surface temperatures are also increasing in the Arctic [6,7,8], and the mechanisms are still unclear. It is consequently beneficial to investigate solar radiation at typical sites in polar regions. Solar energy triggers changes in many atmospheric gases, liquids and particles (GLPs), especially those involving chemical and photochemical reactions (CPRs) in UV and visible (VIS) spectral regions [9,10,11,12,13,14,15,16,17].

Numerous radiative transfer models and empirical models, extensively used in estimating global, direct and diffuse solar radiation at the ground, are briefly reported in [17,18,19,20,21,22,23,24,25,26]. Satellite-retrieved upward and downward surface global irradiances were found to be underestimated by 74.0% in 2000–2018, and need to be validated by using surface measurements [27]. Therefore, in model development and application, in-situ measurements of solar radiation at the surface are mostly used. However, accurate solar radiation measurements are only possible in a very small number of manned stations, and are expensive and highly demanding. Considering the very large uncertainty in satellite estimations, parameterizing an empirical model to estimate global solar radiation (G) starting from real data (solar radiation and meteorological parameters) can be useful, and helps to increase spatial coverage in an area as challenging for operations and observations as Antarctica. The aims of this paper are to investigate (1) the features of global solar radiation (G) at the surface, (2) the losses of G in the atmosphere due to absorbing and scattering processes, and their relative contributions to total loss, (3) albedos at the TOA and at the surface, (4) the relationships between absorbing and scattering radiation and their affecting factors and (5) long-term changes in the above parameters.

An empirical model of global solar irradiance (EMGSI) was parametrized to estimate global solar irradiance at Dome C based on solar radiation and meteorological measurements [17,27]. This model was used to fulfill the above aims, and the results at Dome C were compared with those at Sodankylä (67.367° N, 26.630° E, 184 m) in the Arctic and a site at mid-latitude in the northern hemisphere, Qianyanzhou (26°44′48″ N, 115°04′13″ E, 110.8 m, a subtropical *Pinus* forest, China) (Section 3 and Section 4). The results aim to contribute to a deeper understanding of the basic characteristics and mechanisms in the atmosphere-land system, as well as of regional climates and climate change.

## 2. Data and Methodology

### 2.1. Measurements and Data Selection

Dome C hosts a site of the Baseline Surface Radiation Network (BSRN), a reference network of the Work Climate Research Program (WCRP), endorsed by the Global Climate Observing System (GCOS). The BSRN provides surface-based, high-quality reference measurements of the solar and infrared irradiance and radiation balance [28,29,30]. Solar radiation [31,32] and meteorological variables [33] were measured at the Dome C (75°06′ S, 123°21′ E, 3233 m) station, located at the Antarctic interior plateau [29,30,34] (Table 1). The data from January 2006 to December 2016 are considered in this study. Global solar irradiance (G) and diffuse horizontal irradiance (S) were measured by secondary standard un-shadowed and shadowed pyranometers, respectively (model CM22, Kipp & Zonen Inc., Delft, The Netherlands). Direct beam irradiance (D) was measured by a pyrheliometer (CH1, Kipp & Zonen Inc., Delft, The Netherlands). Following the BSRN protocols, all radiation sensors were usually calibrated every 2 years. At Dome C, different sensors were used in rotation between every 2–4 expeditions after their traceable calibration was performed at the World Radiometric Reference (WRR), which is maintained at the World Radiation Center (WRC) in Davos, Switzerland. Further details are described in [30]. Meteorological variables, such as air temperature (T), relative humidity (RH) and wind speed (v), were obtained from the IPEV/PNRA Project “Routine Meteorological Observation at Station Concordia”—http://www.climantartide.it (accessed on 17 January 2022). The surrounding areas of the Dome C station are covered by a homogeneous snow surface with a low slope (<0.2°) ([35], and references therein). The air temperature varies seasonally from −80 °C to −20 °C [36]. The mean temperature is −20 °C in summer and −70 °C in winter, and the maximum wind speed is 18 m s^−1^ [36]. A minimum surface temperature of −98 °C was observed during the winters of 2004–2016 [37]. The atmosphere is cold, dry, clear and clean [38]. The aerosol optical depth (AOD) in Antarctica remained at a stable low level of 0.022 during 1996–2013 and 0.024 during 2006–2019 [39,40]. The atmosphere over the Antarctic Plateau is slightly contaminated by aerosols [41,42].

To ensure reliable data, the hourly global solar radiation measured as larger than 20 W m^−2^ was used in the analysis, including daily and monthly averages. The extreme hourly irradiances (G, S, D) and S/G were removed, and similar data criteria were applied, which were also used in other studies [27,43]. In addition, when G is < 20 W m^−2^, the sun is very low, causing larger observational errors in S/G. Hourly solar irradiance and meteorological parameters during 2008–2011 were first selected to develop an empirical model of solar global irradiance (EMSGI) under all-sky conditions. The model then was applied to estimate G and its loss in the atmosphere, and the albedos at the TOA and the surface (referred to as TOAsur) over the whole 2006–2016 period.

### 2.2. Model Formulation, Development and Evaluation

**Figure 1:**
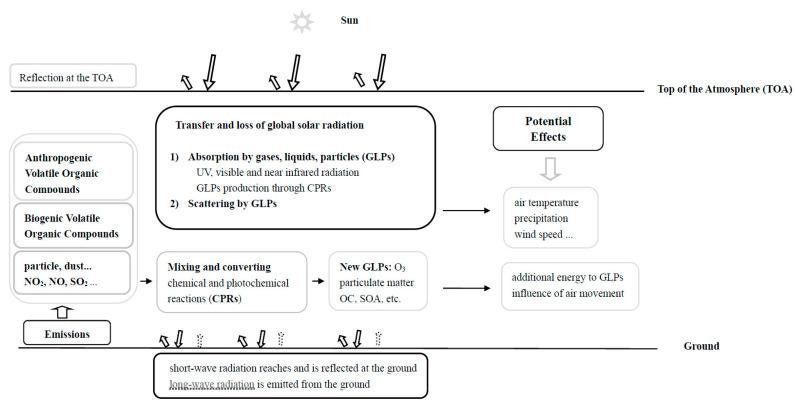
Flow chart of the global solar radiation transfer and loss in the atmosphere and their potential effects.

Figure 1 shows a flow chart of the transfer and loss of global solar radiation in the atmosphere and their potential effects. Solar radiation interacts with atmospheric GLPs through two key processes, i.e., (1) absorbing and (2) scattering. They are taken into consideration in our parametric model as follows [17]: (1) in the photochemical term, the effective absorption of G by GLPs is calculated by means of an extinction term such as e^−kWm^ × cos(Z), where k is the mean absorption coefficient of water vapor, W is the water vapor content in the atmospheric column (cm), m is the optical air mass and Z is the solar zenith angle. The water vapor content is estimated following [44], with W = 0.02 × E × 30, where E is the water vapor pressure at the surface (hPa). The meaning and mechanism of this term in the short wavelength region (i.e., UV, VIS and near infrared, NIR) are fully reported in [17], emphasizing GLP absorption and their indirect use in CPRs thorough OH radicals and volatile organic compounds (VOCs). (2) In the scattering term, the total scattering of G induced by GLPs is evaluated by an extinction term dependent on the diffuse ratio (S/G) being e^−S/G^. An EMGSI model under all-sky conditions was optimized for Dome C to estimate the global radiation, G_cal_ [17,27]:

(1) $$G_{\mathrm{cal}}=A_{1}e^{{}^{-\mathrm{kWm}}}\times \cos(Z)+A_{2}e^{{}^{-{S/G}_{\mathrm{obs}}}}+A_{0}$$

where G and S are the hourly global and diffuse horizontal solar energy densities at the surface (MJ m^−2^), respectively. A_1_ and A_2_ (MJ m^−2^) parameterize the amplitude of the absorption and scattering, respectively, while A_0_ (MJ m^−2^) is a negative value parameterizing the reflection of global solar irradiation at the TOA. Equation (1) represents the total solar irradiation at the TOA (A_1_ + A_2_ − A_0_), hence, it should be equal to or close to the solar constant (I_0_ = 1367 W m^−2^, a widely used value, equal to 4.92 MJ m^−2^). It is also recommended that an updated solar constant value of 1361.1 W/m^2^ is used in future calculations [45].

To determine an empirical model that represents a good relationship between physical and chemical processes in a realistic atmosphere, more high-quality data, i.e., 2771 hourly data (Z < 75°, sample number n = 2771) from January, February and October–December (JFOND) of 2008–2011, were used for model development. The usage rate of the observed data was 43.60%. Hourly averages of solar irradiance and meteorological variables (i.e., T, RH, E) were calculated and used to also estimate daily and monthly averages [46].

**Figure 2:**
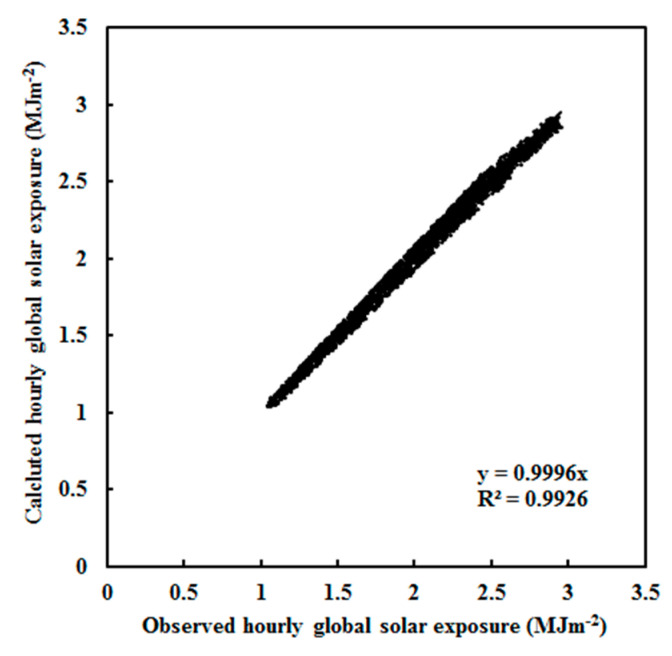
Scatter plot of calculated versus observed hourly global solar exposure under all-sky conditions at Dome C (n = 2771).

All coefficients in Equation (1) were obtained by using a multi-parameter fit of the observed hourly global solar exposure, i.e., by analyzing 2771 pieces of hourly data of G, S/G and E, as well as Z, to determine all coefficients. The results are presented in Table 2, including the optimized parameter A_i_, the coefficient of determination (R^2^), the mean absolute value of relative error (δ) between calculated and measured G, the mean absolute deviations (MAD, in exposure unit, MJ m^−2^, and as a percentage of the mean measured value, %), and the root mean square errors (RMSE, in exposure unit and as a percentage of the mean measured value). Figure 2 shows a scatter plot of calculated versus observed G. The slope of the linear regression of G_cal_ on G_obs_ is 0.9996 with an R^2^ of 0.9926, which is different from R^2^ in Table 2 because different equations were used (the linear regression uses G_cal_ = 0.9996 × G_obs_ and Equation (1)). The calculated G is in good agreement with the measured G under all-sky conditions.

Based on the analysis of hourly data (n = 2771), we obtained the following results: There was a strong correlation between G and the absorbing and scattering terms (R = 0.996, at the confidence level α = 0.001). The correlation between G and the absorption term e^−kWm^ × cos(Z) (R = 0.996) was stronger than the correlation between G and the scattering term e^−S/G^ (R = 0.594), while a weak correlation existed between the absorption and the scattering terms (R = 0.575). This shows that the absorbing and scattering processes are rather well and separately accounted for by the EMGSI model and Equation (1). The RMSE (0.043, Table 2) was less than the mean RMSE (0.22) calculated using 7 independent a-priori models with better estimations out of the 105 empirical models [23], showing that the EMGSI model performs reliable simulations. The calculated monthly average of G was also in line with the observations, with a relative bias of 1.30% for the average values and 2.63% for the maximum values. The RMSE values were 0.03 MJ m^−2^ and 1.15%. The above corresponding values for mean annual G were 1.30%, 1.64% (relative bias), 0.03 MJ m^−2^ and 1.60% (RMSE).

**Figure 3:**
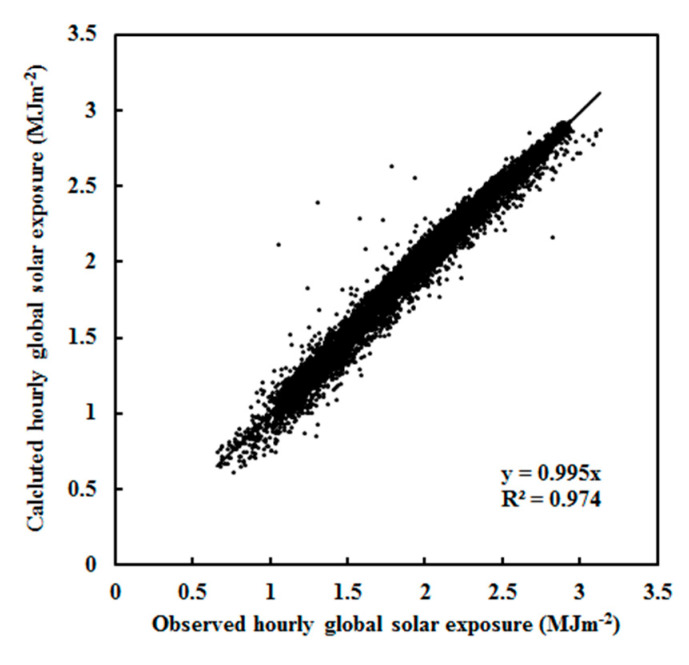
Scatter plot of calculated versus observed hourly global solar exposure under all-sky conditions at Dome C (n = 6356) over the optimization period 2008–2011.

**Figure 4:**
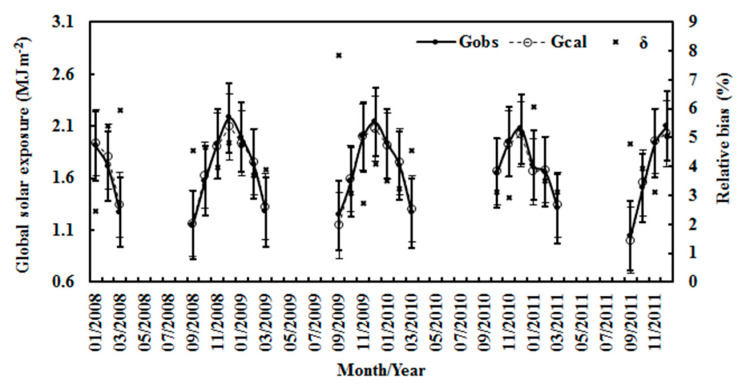
The observed and calculated monthly averages of hourly global solar exposure (G_obs_ and G_cal_) and relative biases (δ) in all-sky conditions at Dome C. The error bars show the standard deviations of the observed and calculated values (1σ, black and bold for the calculated values).

**Figure 5:**
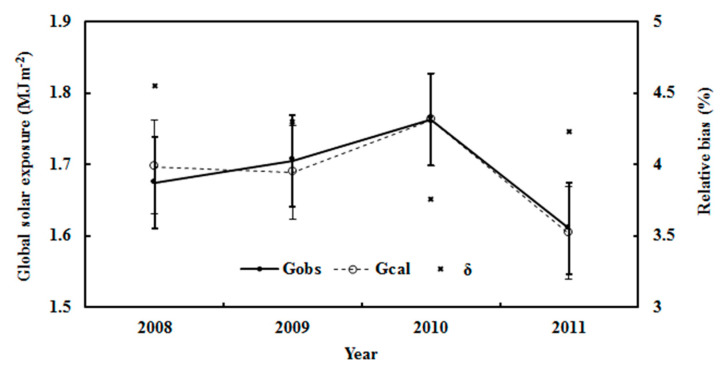
The observed and calculated annual averages of hourly global solar exposure (G_obs_ and G_cal_) and relative biases (δ) in all-sky conditions at Dome C for the period used to accomplish the optimization of the EMGSI model. The error bars show the standard deviations of the observed and calculated values (1σ, black and bold for the calculated values).

Measurements of global solar irradiance for Z < 75° in January–March and September–December during 2008–2011 were used to validate the EMGSI model. The mean absolute relative bias was 4.03%, and the NMSE was 0.002. The RMSE was 0.08 MJ m^−2^ and 4.72% (n = 6356). Figure 3 shows a scatter plot of the calculated vs. observed global exposure. Considering that the uncertainties of numerous solar radiation models are well up to 20% [47], the extremely clean atmosphere (i.e., mean S/G = 0.261) over Dome C guarantees better performances for the model in the simulation of G. The calculated monthly average of G was also in agreement with the observed G, with a relative bias of 4.22% for the average and 7.85% for the maximum. The RMSE values were 0.08 MJ m^−2^ and 5.00%. The standard deviations of the calculated and observed global solar exposures were 0.316 and 0.333 MJ m^−2^ (Figure 4). The annual average of the estimated and the observed G varied in patterns similar to the relative bias, with 4.20% for the average and 4.54% for the maximum. The RMSE values were 0.03 MJ m^−2^ and 5.65%. The standard deviations of the calculated and observed global solar exposures were 0.065 and 0.064 MJ m^−2^ (Figure 5). Both the calculated and measured G decreased by 1.18% and 0.78% per year, respectively, during 2008–2011.

Based on the above results, the empirical model showed a rather good performance in simulating hourly, monthly and annual global solar irradiance under all-sky conditions.

## 3. Results

### 3.1. Global Solar Radiation during 2006–2016

To investigate the basic features of G and the meteorological variables at Dome C, observed hourly data from 1 January, 2006 to 30 November, 2016 were used—considering only the months from September to April, and excluding the polar night from the analysis. From 2006–2016, the averages of observed hourly G, S and D (n = 33311) were 1.34 (corresponding to 371.53 Wm^−2^), 0.34 (94.12 Wm^−2^) and 0.99 (277.40 Wm^−2^) MJ m^−2^, respectively. The direct horizontal radiation dominated G and contributed to 74.67% of it, while the diffuse solar radiation contributed to 25.33%. The mean S/G was 0.308, and the averages of T, RH and E were −42.0 °C (ranged from −15.8 to −79.9 °C), 57.06% and 0.18 hPa, respectively. The average air pressure (p) and v were 645.04 hPa and 6.66 ms^−1^, respectively.

The hourly G was calculated for Dome C for the 1 January 2006–30 November 2016 period, using the empirical model of global solar irradiance and its input parameters (the observed hourly global and diffuse solar irradiance for the S/G factor and E). We only considered observed hourly G > 20 W m^−2^ where the solar zenith angle was lower than 75°. The estimated and observed hourly global solar exposures varied similarly, and the estimated values were lower than the observed by 18.40% on average: the NMSE was 0.013 (MJ m^−2^), and the RMSE values were 0.146 MJ m^−2^ and 10.90% in 2006–2016. These values were a little larger than the corresponding ones used in model development and validation (n = 2771, 6356). This is acceptable considering that the empirical model describing the global solar irradiance and its relationships with the absorbing and scattering processes are determined at optimal atmospheric conditions (i.e., clean atmosphere, low S/G at 0.261 during 2008–2011 in the model development). In contrast, (1) the relative error of 18.40% was less than the 20% uncertainty of popular solar radiation models [47], and (2) the RMSE value of 0.146 (MJ m^−2^) was smaller than the 0.22 obtained using the 7 models with better performances out of the 105 empirical models [23].

**Figure 6:**
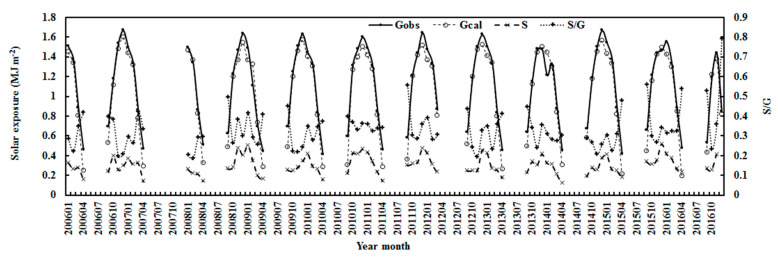
Monthly calculated and observed global solar exposures (G), observed diffuse exposure (S) and scattering factor (S/G) at Dome C.

The calculated and observed monthly global exposure, diffuse exposure and S/G are shown in Figure 6. Generally, the global solar exposure displayed strong seasonal variations and peaked in December (e.g., 2006, 2008, 2011, 2014). The diffuse horizontal radiation followed a similar variation pattern to G, and was influenced by the scattering substances (reflected in S/G). The diffuse ratio of S/G, used in the scattering term of Equation (1), didn’t show evident seasonal variations and frequently peaked in April and September.

Over the 11 years, the monthly mean observed and calculated G decreased by 0.018% and 0.001% per month, respectively, while the observed diffuse irradiance S increased by 0.11% per month. They were associated with the increases in S/G by 0.17% and in E by 0.25% per month. The air temperature and relative humidity increased by 0.02% (corresponding to 1.22 °C) and 0.10% per month, respectively. On average, the annual air temperature increased by about 1.22 °C during 2006–2016, according with the general Antarctic warming [48]. The monthly mean T, RH and E displayed synchronous variations, with strong correlations between T and RH and between T and E (R = 0.923 and 0.900, respectively). The above correlation coefficients were 0.903 and 0.974 for the annual averages. 

**Figure 7:**
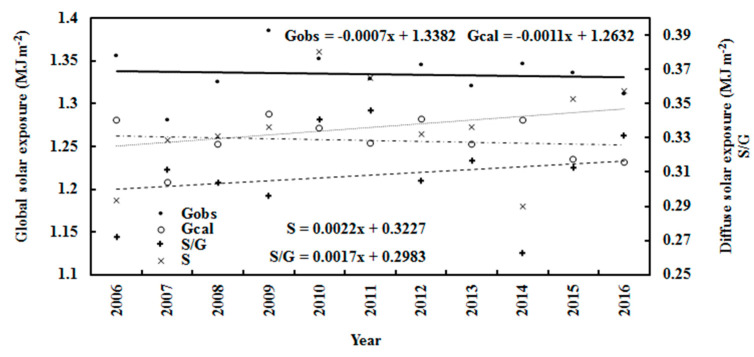
Annual calculated and observed global solar exposures (G), observed diffuse exposure (S) and scattering factor (S/G) at Dome C, together with their trend lines.

**Figure 8:**
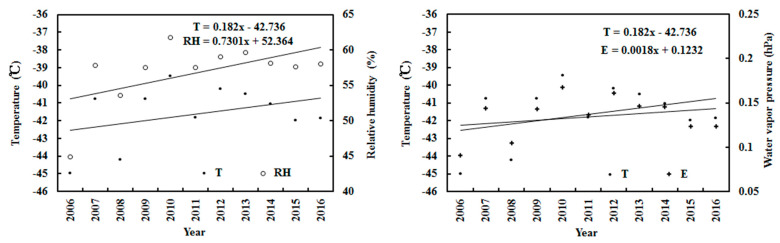
Annual air temperature (T), relative humidity (RH) and water vapor pressure (E) at Dome C.

On an annual basis, over the 11 years, the annual mean of G_obs_ and G_cal_ decreased by 0.05% and 0.09% per year, respectively, and D increased by 0.68% per year (Figure 7). They were associated with annual increases in S/G of 0.57% and in E of 1.46%. The air temperature and relative humidity increased by 0.43% (corresponding to 1.80 °C) and 1.39% per year, respectively. In general, the annual air temperature increased by 1.80 °C (Figure 8), demonstrating the warming climate of the Antarctic Peninsula during the 2006–2016 period [48]. This was similar to the Arctic warming at Sodankylä, which had an annual air temperature rise of 2.09 °C during 2000–2018 [27]. Over the 11 years, the annual mean calculated and observed global irradiances at Dome C were 1.05 and 1.12 MJ m^−2^, corresponding to 291.52 and 311.48 W m^−2^, respectively, indicating that a small part of the total global solar radiation (G/I_0_), 21.33% and 22.79%, arrived at the surface. The annual mean calculated and observed global irradiances were clearly attenuated by the atmospheric substances and inversely varied with the scattering factor S/G (Figure 7). The correlations between G_cal_ and S/G and between G_obs_ and S/G were 0.450 and 0.338, respectively.

To better understand the average environmental conditions at Dome C during 2006–2016, annual averages were calculated and found to be 316.49 and 84.78 Wm^−2^ for global and diffuse solar radiation, 0.332 for S/G and −46.23 °C, 52.00% and 0.10 hPa for T, RH and E, respectively.

### 3.2. The Losses of Global Solar Radiation in the Atmosphere during 2006–2016

**Figure 9:**
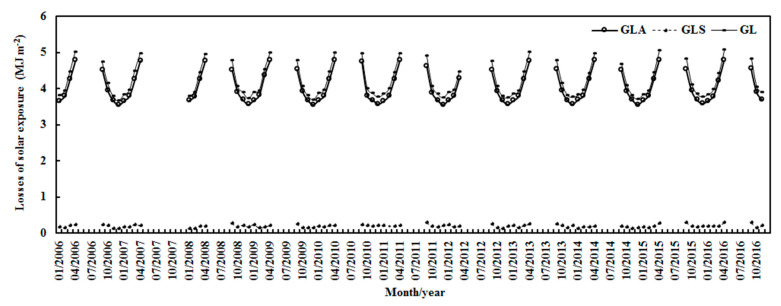
Monthly losses of global solar exposure due to absorbing and scattering substances (G_LA_, G_LS_) and total losses (G_L_ = G_LA_ + G_LS_) at Dome C.

The hourly losses of G due to the absorbing and scattering substances (G_LA_, G_LS_) were estimated by the terms A_1_(1 − e^−kWm^ × cos(Z)) and A_2_(1 − e^−S/G^), respectively, while their sum provides the total loss, G_L_ = G_LA_ + G_LS_. On a monthly basis, G_LA_ caused by absorbing substances dominated the total loss and displayed clear seasonal variations. The lowest values were observed in December, and the higher values in April and September. G_LS_ caused by scattering substances also exhibited clear seasonal variation, with peaks in November and February. From January 2006–November 2016, (1) the monthly G_LA_ decreased slightly by 0.005% (or kept stable), associated with an increase in water vapor of 0.252%; (2) the monthly G_LS_ increased by 0.054%, associated with an increase in S/G of 0.168%; and (3) the monthly G_L_ decreased by 0.002% (or kept stable, Figure 9).

**Figure 10:**
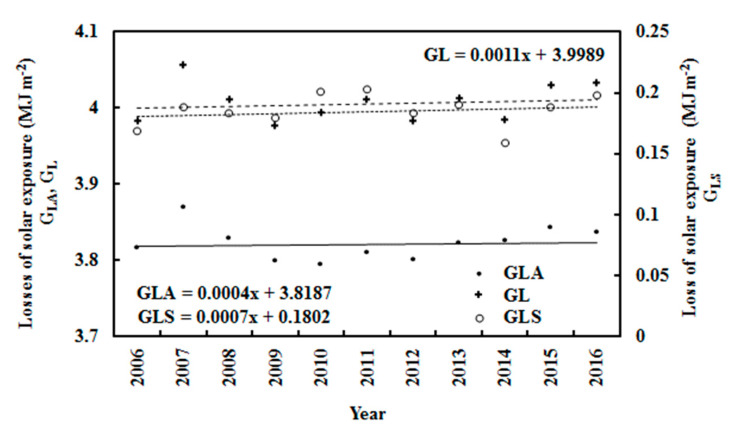
September to April (annual) losses of global solar exposure caused by absorbing and scattering substances (G_LA_, G_LS_) and total losses (G_L_ = G_LA_ + G_LS_) at Dome C.

The annual (i.e., September–April) losses of G in 2006–2016 are shown in Figure 10. The absorbing, scattering and total losses show interannual behavior. This was also observed in the variations of T, RH and E, and especially RH. G_LA_ increased by 0.01% per year, associated with an increase in E of 1.46%; G_LS_ increased by 0.39% per year, associated with an increase in S/G of 0.57%; and annual G_L_ increased by 0.28% per year.

**Figure 11:**
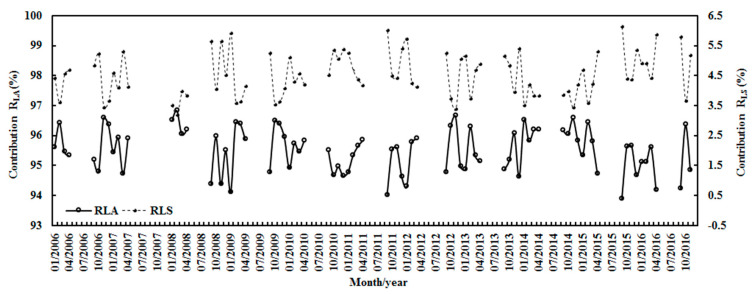
Contributions (R_LA_, R_LS_) of monthly absorbing and scattering losses to the monthly total loss at Dome C.

From January 2006–November 2016, the average contributions of monthly absorbing and scattering losses (R_LA_ = A_1_(1 − e^−kWm^ × cos(Z))/(A_1_e^−kWm^ × cos(Z) + A_2_e^−S/Gobs^), R_LS_ = A_2_(1 − e^−S/Gobs^)/(A_1_e^−kWm^ × cos(Z) + A_2_e^−S/Gobs^)) to the total loss were 95.49% (in the range of 93.87–96.84%) and 4.51% (3.16–6.13%), respectively (Figure 11). This corresponds to the monthly means of E at 0.09 hPa (0.00–0.38), S/G at 0.326 (0.184–0.559) and T at −46.43 °C (−28.75–−65.13 °C). In general, R_LA_ was higher in October–February and lower in April or September, indicating that the absorption mechanism dominates the attenuation of G. R_LS_ varied inversely compared to R_LA_ (i.e., most peaks appeared in April or September, with lower values from October–February). The above corresponding absorbing and scattering losses (R_LA_, R_LS_) were 95.46% (95.04–96.08%) and 4.54% (3.92–4.94%) for the annual average, respectively, and the annual averages of E, S/G and T were 0.13 hPa (0.09–0.17), 0.308 (0.262–0.345) and −46.43 °C (−28.75 to −65.13 °C), respectively.

From 2006–2016, the annual average monthly loss of G_LA_, G_LS_ and G_L_ was 4.02 (3.53–4.78), 0.19 (0.12–0.30) and 4.21 (3.67–5.07) MJ m^−2^, respectively, and the average reflection at the TOA was 1.10 MJ m^−2^, corresponding to 1116.58, 53.58, 1170.17 and 304.79 W m^−2^, respectively. It is implied that the higher energy related to G_LA_ results in larger changes in the air temperature than for G_LS_ (see Section 3.3 and Table 5).

### 3.3. Global Solar Radiation and Its Loss in the Atmosphere in the Period from October to March (2006–2016)

To explore the characteristics and mechanisms of solar radiation, regional climates and their interactions, we computed monthly averages of G and the absorbing and scattering factors, as well as of E, the diffuse ratio (S/G) and the meteorological parameters (T, p, v). We then calculated their cross correlations, limiting the analysis to the period from October to March during 2006–2016. The data of April and September were not considered, because of the lower solar radiation and therefore the reduced number of samples. Furthermore, solar radiation and meteorological variables were analyzed for two situations: solar altitude angles (h) larger than 5° and 10°, respectively.

The calculated global solar radiation also exhibited good performance. Table 3 shows the average performance of the model in simulating G_cal_ based on selected statistical metrics as computed from the available monthly values (n = 62). Generally, the calculated global solar exposure was in good agreement with that observed, and an even better performance was obtained for the situation h ≥ 10° than for h ≥ 5°. This can be attributed to (1) the lower uncertainties in radiation measurements and air-mass calculations, and (2) the much cleaner atmosphere, i.e., a lower S/G (0.268) for h ≥ 10° compared to an S/G = 0.296 for h ≥ 5°. For both situations, the water vapor pressure was at the same level, 0.129 and 0.120 hPa, respectively.

Variation trends in the monthly mean solar radiation and meteorological variables are given in Table 4. For the two situations h ≥ 5° and h ≥ 10°, the observed and calculated monthly global solar exposure, as well as the observed diffuse exposure, increased, the monthly losses of G_LA_ and G_L_ decreased and G_LS_ increased. T, RH and E increased. In more detail, there was a little larger air temperature increase (about 0.29 °C) for the situation h ≥ 10° compared to h ≥ 5°. Air temperature increases were mainly caused by the increases in global and diffuse solar radiation at the surface and scattering loss. Scattering processes/energy (diffuse radiation and scattering loss) therefore played a positive role in climate warming at Dome C during the 11 years, although the small effects of other factors in the changes between h ≥ 10° and h ≥ 5°, such as air advection and cloud amount changes, should also be considered.

To comprehensively investigate the interactions and mechanisms between solar radiation and atmospheric parameters, correlations among the above variables were calculated (Table 5). Strong correlations were found: between T and observed and calculated G; between T and the absorbing and total losses of global solar exposure (G_LA_, G_L_); between G_LA_ and G_L_ and E; between scattering loss (G_LS_) and S/G; and between p and E. These correlations indicate that (1) air temperature is evidently influenced by G at the surface, especially by absorbing energy lost in the atmosphere. (2) Absorbing and scattering substances (described by E and S/G) play important roles in the absorbing and scattering mechanisms, respectively. (3) Water vapor in the whole atmospheric column plays a more important role in air pressure than scattering substances. (4) Absorbing lost energy into the atmosphere contributes significantly more to air temperature (representing atmospheric internal energy) and its change than scattering energy. These features are more evident for the situation h ≥ 10° than for h ≥ 5°. Strong correlations existed between T and E, measured as 0.915 and 0.917 for the situations h ≥ 5° and h ≥ 10°, respectively.

Monthly and annual averages of solar radiation and meteorological parameters were also calculated for h ≥ 5° and h ≥ 10° (Table 6). In general, the monthly and annual averages of all parameters were close for h ≥ 5° or h ≥ 10°. The larger G at the ground corresponded to a higher T (as well as higher humidity and water vapor) for h ≥ 10° compared to h ≥ 5°, indicating that the G arriving at the surface plays a significant role for the mean T, as well as for the atmospheric internal energy. The contributions of the absorbing and scattering losses to the total loss were similar for the monthly and annual averages under the two situations, and were about 96% and 4%, respectively. There were strong correlations between the monthly mean G_L_ and G_LA_ (R = 0.994, 0.995) and a weak correlation between G_L_ and G_LS_ (R = 0.336, 0.294) for the situations h ≥ 5° and h ≥ 10°, revealing that the absorbing loss due to absorbing substances dominates the variation patterns of the total loss of G.

### 3.4. Sensitivity Study

**Figure 12:**
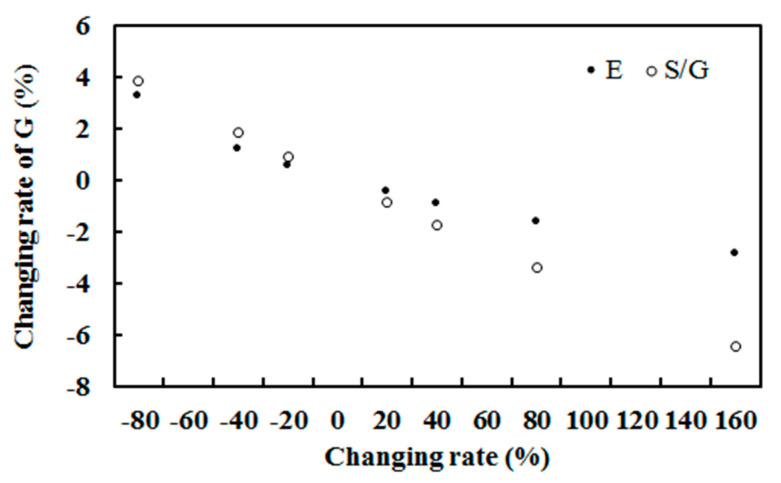
Change rate of G (%) due to changes in E or S/G (%), with S/G and E retaining their original values.

The response of the estimated hourly global solar exposure to changes in the atmospheric absorbing or scattering substances (represented by E and S/G) was studied using Equation (1) (n = 2771), while the other factors remained at their original values. The results are presented in Figure 12 and Table 7.

The global solar exposure at the ground increased/decreased with the decrease/increase of water vapor, indicating that an increase in absorbing substances gives rise to the more attenuation of global solar radiation in the atmosphere and less G arriving at the ground. The global solar exposure also increased/decreased with the decrease/increase in scattering substances, displaying that an increase in scattering substances also results in a large loss of global solar radiation. The global solar exposure was more sensitive to changes in the scattering factor (S/G) than the absorbing factor (E). The changes in scattering substances (clouds, aerosols, SOA, etc.) seem to have stronger effects on global solar radiation than the absorbing substances. For example, the averaged ratio between the response rate of G to S/G and that of G to E at different changing rates was about 1.63 (from 0.57 to 2.27). The responses of global solar radiation to changes in both E and S/G were negative and nonlinear (Figure 12, Table 7).

### 3.5. Albedos at the TOA and the Surface

Reflections from the TOAsur are important factors influencing radiative transfer, energy balance and climate. They should be thoroughly explored in the study of climate change [49,50,51,52]. For albedo estimations and evaluations we used the monthly shortwave flux and incoming solar flux at the TOAsur for all skies, clear skies (cloud free) and clear skies over a 1° × 1° region (https://ceres.larc.nasa.gov/products.php?product=EBAF-Product, accessed on 17 January 2022). The data were obtained from the Clouds and the Earth’s Radiant Energy System (CERES) Energy Balanced and Filled (EBAF) Edition 4.1 [53,54].

It should be emphasized that there is an important homogeneous and unique snow surface featuring a strong reflection at Dome C. An algorithm developed for albedo calculations at the TOA and the surface for Sodankylä and QYZ [17,27] has been adapted for Dome C. Albedo is assumed to be isotropic at the TOAsur; A_0_ represents the overall contribution of the coupled surface-atmosphere at the TOA, whereas the sum A_1_ + A_2_ + A_0_ represents I_0_. Thus, the albedos at the TOAsur (Albedo_TOA_, Albedo_Sur_) were estimated using Equations (2) and (3), respectively:

Albedo_Sur_ = (A_0_/Tran_sca_ + A_2_/Tran_sca_ + A_1_ × Tran_abs_ × A_2_/(A_1_ + A_2_))/(A_1_e^−kWm^ × cos(Z) + A_2_e^−S/Gobs^) (2)

Albedo_TOA_ = (A_0_ + A_2_ + A_2_ × Tran_sca_ × Albedo_Sur_ + A_1_ × A_2_/(A_1_ + A_2_) + A_1_ × Tran_abs_ × Albedo_Sur_)/(A_1_ + A_2_) (3)

where Tran_abs_ and Tran_sca_ are the mean transmittances of absorbing and scattering exposures in the atmosphere calculated by e^−kWm^ × cos(Z) and e^−S/Gobs^, respectively. In detail, the reflections at the surface were contributed by individual reflections and scattering derived from the TOA (A_0_/Tran_sca_, A_2_/Tran_sca_), while A_1_ × Tran_abs_ × A_2_/(A_1_ + A_2_) is the scattering contribution from the absorbing process. The reflections at the TOA were contributed by reflection A_0_, scattering A_2_ (considering that the scattering is isotropic at the TOA), scattering from the reflection at the surface (A_2_ × Tran_sca_ × Albedo_Sur_) and scattering contributed from the absorbing at the TOAsur (A_1_ × A_2_/(A_1_ + A_2_), A_1_ × Tran_abs_ × Albedo_Sur_).

**Figure 13:**
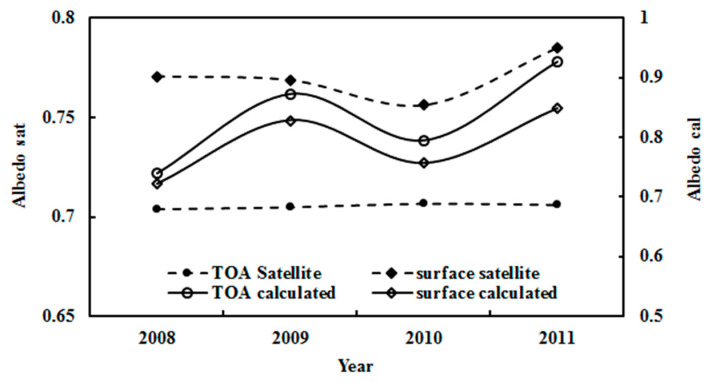
Annual mean albedos averaged in JFOSD over the period 2008–2011 calculated (Albedo cal) and satellite-retrieved (Albedo sat) under clear-sky conditions at Dome C.

The calculated averaged albedos at the TOA and the surface in the months JFOND (n = 2771) during 2008–2011 were 0.728 and 0.739, respectively. The corresponding satellite-derived albedos were 0.686 and 0.770 under clear-sky conditions, respectively. The estimated albedos were in good agreement with the satellite measurements, with relative biases of 6.08% and 4.08%. Similarly, the annual average albedos in JFOND from 2008 to 2011 was also computed using hourly observational data, and were found to be 0.743, 0.873, 0.795 and 0.926 at the TOA, and 0.722, 0.828, 0.757 and 0.848 at the surface (Figure 13). These albedos corresponded to the satellite values under clear-sky conditions with relative biases from 5.21% to 31.16% at the TOA and from −9.29% to 5.52% at the surface. The satellite- and model-estimated albedos exhibited similar variational tendencies during 2008–2011, i.e., the albedos at the TOAsur were increased by 0.41% and 0.13% for the satellite-derived estimates, and 4.33% and 6.73% for the empirical model estimates, respectively.

To thoroughly investigate the albedos at the TOAsur, the annual mean albedos in JFOND from 2008–2011 under all-sky conditions was calculated at 56.48% and 83.68% when h ≥ 5°, and 58.27% and 75.86% for h ≥ 10°, respectively. In comparison, the TOA and surface albedos retrieved from the satellite were 70.52% and 79.90%, respectively. Generally, the estimated albedos at the TOAsur agreed with the satellite observations, with relative biases of −19.88% and 4.73% at the TOAsur for h ≥ 5°, and −17.34% and −5.05% for h ≥ 10°, respectively. The larger relative biases at the TOA were related to (1) the time and space match and (2) the limited overpass time for the satellite and continued and reliable observations for absorbing and scattering GLPs (E, S/G). The model-estimated albedos can capture more detailed features, e.g., monthly and annual variations.

**Figure 14:**
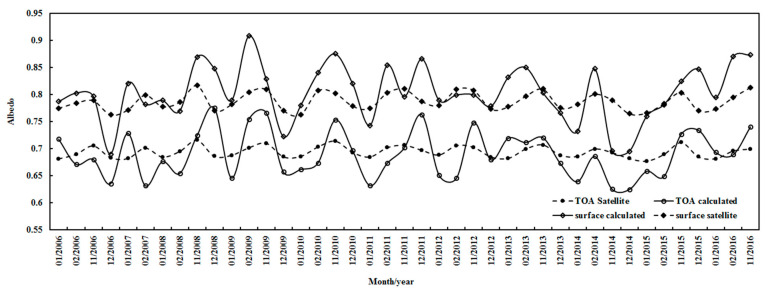
Monthly mean albedos calculated and satellite retrieved at the TOA and the surface under all-sky conditions at Dome C in the period 2006–2016.

Using Equations (2) and (3) and hourly observational data with h ≥ 10°, the monthly mean albedos at the TOAsur were computed for all-sky conditions during 2006–2016 (Figure 14). The calculated albedos at the TOAsur exhibited clear month-to-month variations, and corresponded to the satellite-derived values at the TOAsur. The monthly mean ratios of calculated to satellite-derived albedos were 0.99 (0.90–1.13) and 1.02 (0.88–1.13) at the TOAsur, respectively. From 2006–2016, both calculated and satellite-derived monthly albedos decreased by 0.01% at the TOA, and increased by 0.01% at the surface.

**Figure 15:**
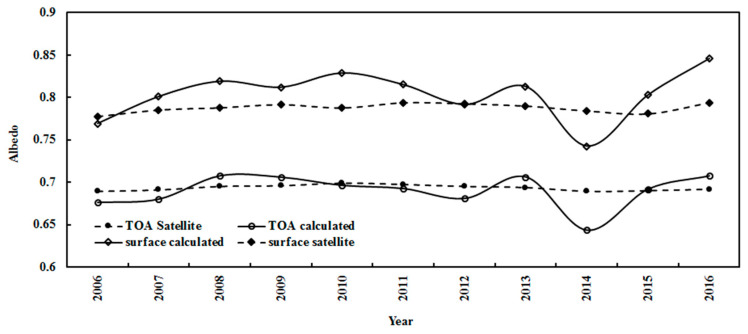
Annual mean calculated and satellite-retrieved albedos in JFND at the TOA and the surface under all-sky conditions at Dome C in the period 2006–2016.

The annual albedos in JFND at the TOAsur were also estimated using monthly values, and agreed with the corresponding values from the satellite data (Figure 15). The annual mean ratios of calculated to satellite-derived albedos were 1.00 (0.93–1.02) and 1.02 (0.95–1.07) at the TOAsur, respectively. The error of the retrieved albedos using MODIS data in the shortwave region is reported as 85.9% [55]. Both calculated and satellite-retrieved annual albedos decreased slowly by 0.001% and 0.004% per year at the TOA, respectively, and increased by 0.14% and 0.06% per year at the surface, respectively. The annual averaged albedos in JFND were 0.690 (0.644–0.707) and 0.804 (0.742–0.846) at the TOAsur for the calculated albedos, and 0.694 (0.690–0.699) and 0.788 (0.778–0.794) for the satellite-derived. Generally, the estimated albedos showed good accuracy. Both calculated and satellite-retrieved albedos exhibited similar characteristics, e.g., the albedos at the surface were larger than those at the TOA, indicating a strong reflection from the snow surface.

As a reference, the annual mean values in JFND from 2006–2016 for the atmospheric substances (i.e., S/G) increased by 2.10% per year, the water vapor increased by 3.56% per year, the observed global solar exposure decreased by 0.06% and the diffuse exposure increased by 2.04% per year, while air temperature increased by 2.12 °C.

To understand the basic atmospheric characteristics at Dome C, the annual averaged values in JFND from 2006–2016 were calculated: S/G = 0.262, E = 0.185 hPa, T = −35.41 °C, RH = 63.05% and v = 6.82 ms^−1^.

The albedo decrease at the TOA in 2006–2016 may be caused by (1) increases in absorbing GLPs and scattering GLPs in the atmosphere, and/or (2) the direct absorption and indirect consumption of UV and visible radiation by all kinds of atmospheric constituents when reacting with OH radicals and H_2_O [56]. Both the model-estimated and satellite-derived annual TOA and surface albedos showed larger decreases in 2014 compared to 2013. The observed annual E and S/G decreased, while the estimated and observed annual G increased in 2014 compared to 2013, indicating that the atmosphere was dryer and cleaner, and more snow welted into water (its albedo < 0.10). So, the decreased atmospheric GLPs are the main reason for the decreases of the TOA and surface albedos.

Albedos displayed similar variational trends at the two clean regions, i.e., albedos decreased at the TOA and increased at the surface at Dome C and Sodankylä [27], implying that the atmosphere undergoes similar changes in these two regions in response to increases in atmospheric GLPs, and through atmospheric circulations over long time scales [57,58,59].

The TOA and surface albedos can easily be calculated using the empirical model (i.e., Equations (2) and (3)), and using popular radiative transfer models that need more atmospheric parameters, including aerosol, cloud and water properties [60]. They cannot be obtained using the current empirical models (see Introduction). Combining the annual mean albedos of 0.804 at the surface and the solar global irradiance at the ground from 2006–2016, the calculated and observed annual mean global solar irradiance was 57.14 and 61.05 W m^−2^, respectively (corresponding to 0.21 and 0.22 MJ m^−2^).

## 4. Discussion

### 4.1. Application of the Empirical Model for Global Solar Radiation

The EMGSI model was developed based on the analysis of hourly data from 2008–2011, and then applied to estimate G in 2006–2016. In addition, the observations of G can also be used as a further evaluation of the empirical model. It is a step forward and an innovation to use the empirical model and surface measurements to estimate solar radiation and albedos at both the surface and the TOA, and the loss of solar radiation in the atmosphere. The absorbing and scattering processes in radiation transfer can be separately studied, and used to better study the interactions/mechanisms between solar radiation–GLPs–climate change.

According to good estimations of G and albedos at the TOAsur, the empirical model is capable of studying G and related issues, e.g., the interaction of solar radiation—GLPs at Dome C. This empirical model is a further application of previous ones under all-sky conditions, and the mechanism of each term is fully explained in [17,27]. In short, the absorbing term represents the total absorption and use of G caused by all GLPs (1) in the UV region through the OH radicals, H_2_O and BVOCs, (2) in the VIS region through excited NO_2_ (NO_2_*) and (3) in the NIR region through H_2_O, CO_2_, CH_4_ and other GLPs. The scattering term represents multiple scatterings by all GLPs (e.g., aerosols, clouds, fog, rain) in the atmosphere, and multiple reflections between the atmosphere and the surface.

Strong correlations were found between the observed G and the absorbing and scattering terms; their correlation coefficients were 0.988 and 0.435 (n = 33311), respectively. A weak correlation was obtained between the absorbing and scattering terms (R = 0.339). So, the absorbing and scattering terms can be used to generally describe absorbing and scattering processes in the atmosphere at Dome C. Similar features were also found at Sodankylä and Qianyanzhou [17,27]. As absorbing and scattering processes can be separately described, the estimates of the albedos at the TOAsur were well captured (e.g., monthly and annual variations, Figure 14 and Figure 15). The snow surface results in strong reflections and multiple scatterings at Dome C. More studies are needed to capture the fine structure of albedos.

### 4.2. Analysis of the Interactions between Changes in Air Temperature and Solar Radiation

Regional T (or accurately, internal energy of the atmosphere, INEA) change is driven by many factors over a long-term period, but it is more directly and significantly driven by solar radiation (1) attenuated in the atmosphere by all GLPs, and (2) received at the ground, which is converted to long-wave outgoing radiation heating the atmosphere, i.e., the total solar radiation received and accumulated in the atmosphere (part 1 + part 2). To investigate the mechanism associated with the change of T, firstly, hourly solar radiation and meteorological variables were analyzed. The change rates for hourly mean parameters over different time periods, including observed and calculated G (G_obs_, G_cal_), absorbing and scattering losses caused by absorbing and scattering GLPs (G_LA_, G_LS_), total loss (G_L_ = G_LA_ + G_LS_), together with T, E and S/G are presented in Table 8. Generally, T increased, which was associated with an increase in E and atmospheric substances (S/G) for three situations, i.e., optimal atmospheric conditions in 2008–2011 (n = 2771), real atmospheric conditions in 2008–2011 (n = 6356) and real atmospheric conditions in 2006–2016 (n = 33311).The observed and calculated surface G and its losses exhibited different trends, revealing different mechanisms behind air temperature increases (corresponding to the increase of INEA): the increase of T was due to (1) an increase in G at the surface for situation 1, (2) an increase in G at the surface, along with an increase in scattering loss in the atmosphere for situations 2 and 3. In more detail, the increases in INEA and T were contributed by the scattering energy caused by the scattering GLPs, as well as the increase of G at the surface, which turns into long wave radiation heating the atmosphere (and all GLPs). This increased scattering energy was well associated with the increase in the scattering GLPs (S/G) for situations 2 and 3. Secondly, the annual mean change rates (%) calculated using the monthly average in JFND from 2006–2016 were analyzed (Table 9); the increase in T was contributed by the enhanced absorbing and scattering energy and the total loss in the atmosphere, whereas G decreased in this situation. To fully understand the real atmosphere, the main observed parameters are presented (Table 10). A higher T was associated with a larger G at the surface, which was less attenuated by scattering GLPs (situation 1, the cleanest atmosphere), and vice versa (situation 3, the highest GLP load was mainly scattering aerosols, whereas absorbing GLPs were the lowest). In other words, G provides energy to the atmosphere and drives air temperature (INEA) and its increases in different ways, e.g., for three types of interacted states/processes (situations 1–3) between solar radiation and GLPs (Table 10).

There were negative correlations between monthly air temperature and monthly satellite-retrieved albedos at the TOAsur with R = 0.684 and 0.684, respectively, and the corresponding R values were 0.058 and 0.229 for the model-estimated values. However, it should be noted that the air temperature is influenced by many factors, i.e., all solar radiation components, different types of GLPs, and their interactions, and albedo/reflection are also the contributors. The reflection at the TOAsur reduced the solar radiation arriving at the ground that can be converted to long-wave radiation heating the atmospheric GLPs; thus, negative correlations existed between monthly air temperature and albedos at the TOAsur.

The different approaches to how the data are used in the analysis (e.g., hourly or annual averages, and time period) revealed different mechanisms behind the air temperature increase. It is suggested to pay high attention to the data usage. Different types of data were jointly used to explore the mechanisms of climate change thoroughly. It should be mentioned that T and its increase were obviously associated with an increase in E (Table 7, Table 8 and Table 9), revealing that water vapor and other absorbing GLPs (CO_2_, CH_4_, N_2_O, black carbon (BC), organic carbon (OC), some VOCs, etc., play vital roles through the absorption of long-wave radiation emitted from the ground, together with other absorbing GLPs in the UV and VIS regions ([17,56,57] and references therein). In short, the absorbing GLPs directly absorb and/or indirectly use UV and VIS energy through CPRs with OH radicals, NO_2_*, H_2_O, VOCs, etc.; some of this energy is converted to heat energy warming the atmosphere; some GLPs absorb NIR radiation; and some GLPs absorb long-wave radiation converted from the incidents of short-wave radiation at the surface. All these forms of energy heat the atmosphere [17].

It is suggested that the emissions of anthropogenic GLPs should be reduced to slow down air temperature increases and global warming [27]. As the driest and cleanest atmosphere at Dome C (compare S/G at the three sites) provides a unique and good natural laboratory, evident mechanisms of T change and the relations between INEA and solar radiation can be found directly.

Many mechanisms have been proposed (e.g., changing oceanographic or atmospheric circulations in the Introduction), but the reasons for the warming climate at the two poles are still unclear. This study provides another mechanism from an energy source, the sun, and the transfer and distribution/accumulation of its energy in the atmosphere.

### 4.3. Relationship between Wind Speed and S/G

**Figure 16:**
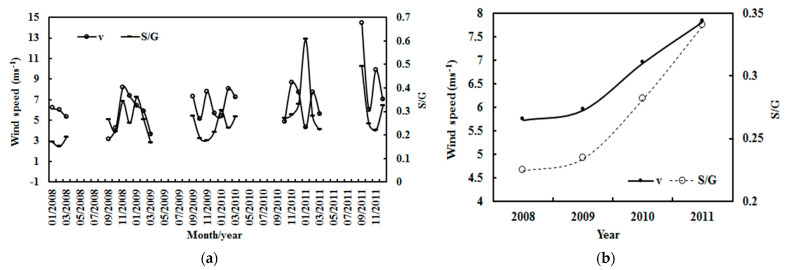
(**a**) Monthly and (**b**) annual wind speed (v) and scattering factor (S/G) from January 2008 to December 2011 at Dome C.

G drives movements in the atmosphere, i.e., atmospheric GLPs, including vertical and horizontal air motions. The relationships between GLP loads and wind speed was investigated using hourly data from 2008–2011 (n = 6356). Some outliers were removed, and 6342 grouped data points were used in the analysis. From 2008–2011, wind speed at Dome C increased by 1.37% for the monthly averages (January–December) and 15.15% for the annual averages (Figure 16). The wind speed showed similar or opposite variations with the S/G for the monthly averages and similar variations for the annual averages. In general, GLPs increased by 0.05% and 23.00% for the monthly and annual averages, respectively, in 2008–2011.

**Figure 17:**
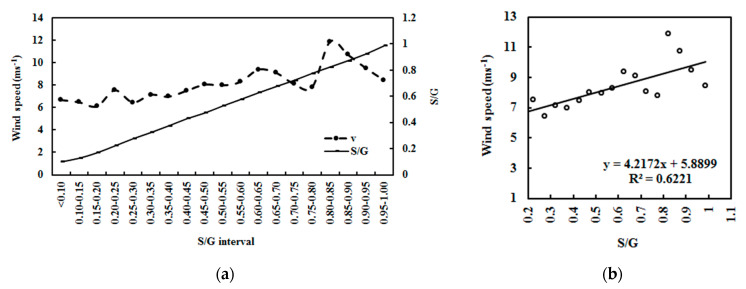
Monthly wind speed (v) and monthly scattering factor (S/G) for *x*-axis using (**a**) S/G interval at 0.05 and (**b**) their scatter plot during January 2008–December 2011.

The increase in v was associated with an increase in S/G for the monthly and annual mean values at Dome C during 2008–2011 (Figure 16). Similar behavior was obviously exhibited in their monthly variations using an S/G interval of 0.05 (Figure 17). These observations reveal that a high v was associated with a high concentration of GLPs (S/G), implying that the increased scattering energy lost in the atmosphere is beneficial to horizontal movements in the atmosphere through an energy transfer from photon energy to kinetic energy. This feature was found because of the very dry and clear atmosphere at Dome C and was supported by observations (e.g., G_LS_ increases at situations 2 and 3). In comparison, other regions with much higher GLP loads (i.e., Sodankylä and QYZ) show strong but opposite relationships between v and S/G [17,27]. So, the large differences in columnar GLP amounts (including components and concentrations) and GLP—solar radiation interactions result in different effects over three typical regions, i.e., different phenomena/mechanisms of climate change (T, v, etc.).

**Figure 18:**
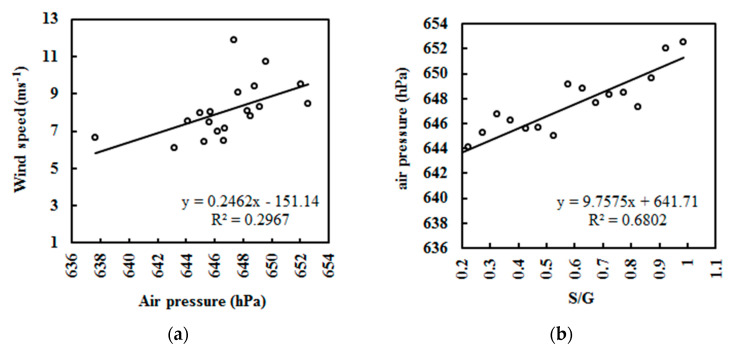
Scatter plot of (**a**) wind speed versus air pressure and (**b**) air pressure versus S/G at different S/G intervals of 0.1 under all-sky conditions at Dome C in the period 2006–2016.

In addition, obvious positive relationships also existed between v versus air pressure *p* and *p* versus S/G at different S/G intervals of 0.1 under all skies (Figure 18); their fitted equations are v = 0.25 × *p* − 151.14 (R^2^ = 0.297, a = 0.001, n = 6342) and *p* = 9.76 × (S/G) + 641.71 (R^2^ = 0.680, a = 0.001, n = 6342), respectively. Thus, the increase in v was mainly caused by an increase in *p*, or more accurately, in atmospheric GLPs. The lower G received at the ground and the lower T make the colder air mass move from the polar point southwards. Observation showed that the average speed direction was 192.3° (n = 6356, median = 190.0°). Thus, gravity plays a dominant role in pushing regional air masses from the south pole to Dome C.

The G lost (by absorption and scattering) in the atmosphere and arriving at the ground displays obvious monthly, annual and interannual variations at Dome C, and drives changes in T and INEA in different ways. It is beneficial to thoroughly analyze the total energy in the atmosphere system and understand the mechanisms of climate warming on regional and global scales.

### 4.4. Comparisons of Global Solar Radiation at Two Pole Sites and a Mid-Latitude Site in 2013–2016

To investigate G and the interactions with its influencing factors, we analyzed and compared G, its loss and other related factors at two polar sites and a mid-latitude site, Qianyanzhou (QYZ). The areas surrounding the Sodankylä and QYZ sites are mainly covered by boreal coniferous and *Pinus* forests, respectively. The annual means of monthly G and other parameters were computed for the three sites under all skies during 2013–2016 (Table 11). The ratios of all parameters between Sodankylä and QYZ (Ratio 1) and Dome C and QYZ (Ratio 2) are also shown in Table 11. The estimated G_cal_ at the surface was 54.23% lower at Sodankylä than QYZ, G_L_ was 63.08% larger at Sodankylä than QYZ, and the albedo at the TOA was 24.05% larger at Sodankylä than QYZ, causing T to be −19.65 °C lower at Sodankylä than at QYZ. Similarly, the above corresponding values were −11.97%, +105.64% and +137.93% at Dome C compared to QYZ. The global solar radiation received at the surface that can be converted to long-wave radiation emitted by the earth’s surface was calculated using G_cal_ × (1-albedo at the surface), and was found to be 0.250, 0.507 and 1.108 MJ m^−2^ for Dome C, Sodankylä and QYZ, respectively, displaying that the energy heating the atmosphere through long-wave radiation emitted from the ground was the lowest at Dome C, followed by Sodankylä and QYZ. One important cause is that the lowest T appears at Dome C.

Comparing the annual contributions to energy losses due to absorption and scattering by GLPs at the three sites, the absorbing substances attenuate G more than the scattering substances (R_LA_ is much larger than R_LS_).

The annual mean E and S/G were the lowest at Dome C, and the highest at QYZ, indicating that the atmosphere is the driest and cleanest at Dome C, followed by Sodankylä with a little more water vapor and GLPs, with QYZ having the highest atmospheric GLP loading. The longer optical length at Dome C and Sodankylä than at QYZ is a prime reason causing the much large losses (G_LA_, G_LS_, G_L_). The annual AOD (aerosol optical depth) at the three poles (Arctic, Antarctic and Tibetan Plateau) are reported at 0.046, 0.024 and 0.098, respectively, with the lowest AOD at the Antarctic [39], corresponding well to the S/G values (Table 11). For example, the ratios comparing the Antarctic to the Arctic were 0.525 for S/G and 0.522 for AOD, respectively. This supports the notion that the scattering factor S/G can describe the scattering substances well, especially aerosols at the two poles.

Change rates (%) of the annual monthly averages of solar radiation and meteorological variables, and calculated albedos at the TOAsur, are reported in Table 12 for the three sites under all-skies during 2013–2016. Decreases in G at the ground and its loss in the atmosphere (G_LA_ kept relatively stable), together with a large increase of albedo at the TOA, led to a decline in T at QYZ (Table 12), indicating that the solar radiation energy that was received by and stayed in the atmosphere clearly plays a dominant and controlling role in the decline in T. A decrease in G at the ground larger than its small increase in losses in the atmosphere caused T to drop at the two poles (Table 12). Therefore, the mechanisms of T change are very complex and depend on changes in the solar radiation components. T and E have strong and positive correlations for h ≥ 5° and h ≥ 10° (Section 3.3), but they did not always change similarly (Table 12). It should be noted that (1) different absorbing and scattering GLPs control solar radiation arriving at the ground and staying in the atmosphere, as well as their distributions (R_LA_, R_LS_) (e.g., for the three typical regions); (2) the amount of GLPs and their changes, together with their interactions with solar radiation components, control the regional climatic mean state and climate change. If changes in the above variables on horizontal and vertical scales exceeded a limit that would prevent the atmosphere from returning back to its previous and normal climate state or the GLP—solar radiation equilibrium state on a timely basis, it would cause an abnormal regional climate and climate change (T, v, precipitation, etc.). The higher number of GLPs there are in the atmosphere, the more abnormal changes and distributions of solar radiation will happen in the horizontal and vertical dimensions and over various regions across the globe, along with more abnormal climatic phenomena. Different chemical compositions (e.g., NO_x_, SO_2_, BVOCs, anthropogenic VOCs (AVOCs)) take part in CPRs, and form a large quantity of secondary products (e.g., O_3_, HCHO, fine aerosols, secondary organic aerosols-SOA). So, much solar UV and VIS radiation is utilized, and their redistribution in the atmosphere and at the surface will change to different extents. Thus, different chemical compositions and concentrations play different roles in solar radiation at the ground and in the atmosphere (Table 11 and Table 12). The high T and E, the high BVOC emissions and O_3_, and the solar radiation in the *Pinus* forest at QYZ [17,61] result in a large SOA and a high number of GLPs (i.e., S/G). All of the above factors contribute to larger increases in albedo at the TOA (11.73%) at QYZ (Table 10 and Table 11).

During 2013–2016, G at the surface decreased at Dome C, which was mainly due to increases in scattering GLPs, losses of G, and albedos at the TOA; G at the surface also decreased at Sodankylä, which was mainly caused by increases in absorbing and scattering GLPs, along with their associated losses in the atmosphere; G at the surface decreased at QYZ, which was mainly due to increases in absorbing and scattering GLPs, absorbing losses, and albedos at the TOA.

In 2013–2016, the largest amount of solar energy (G_L_) was lost in the atmosphere and the largest albedo occurred at the TOA, so, the annual mean T was the lowest at Dome C (−41.39 °C), followed by that at Sodankylä (T = 3.05 °C) and QYZ (T = 22.71 °C). The albedo at the surface mainly depends on the type of surface, e.g., the snow surface has the highest albedo at Dome C, and forest areas have similar albedos at Sodankylä and QYZ. The albedo at the TOA depends on the features of both the atmosphere and the surface, e.g., the largest albedo at the TOA was due to the largest albedo at the surface and then the smallest attenuation by the driest and cleanest atmosphere (E = 0.13 hPa, S/G = 0.31) at Dome C, followed by a much smaller albedo at Sodankylä; the smallest albedo at QYZ was caused by it having the highest absorbing and scattering GLPs (E = 22.38 hPa, S/G = 0.83). The absorbing GLPs play a dominant role in the loss of solar radiation (89.31% vs. 13.69%) at QYZ. The extinction of G is also dominated by absorption in the four seasons for reasons reported in [17].

### 4.5. Normalized Absorbing Energy and Its Potential Effects

**Figure 19:**
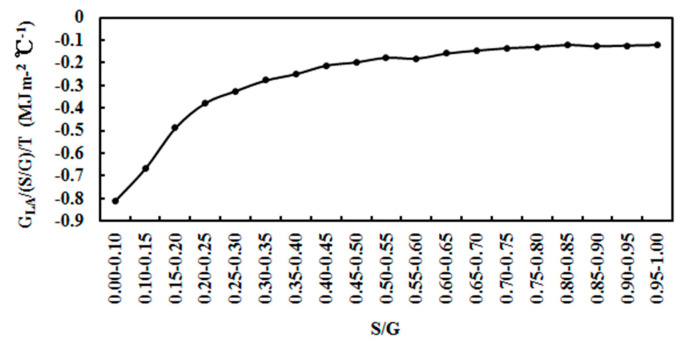
Ratio of solar absorbing loss to S/G and then to air temperature (T) at different S/G intervals at Dome C in the period 2006–2016.

The annual average ratio of absorption loss divided by S/G and then T (G_LA_/(S/G)/T) varied with S/G at an S/G interval of 0.05 (≤1.00) in all skies (Figure 19). The mean ratio (G_LA_/(S/G)/T), or the normalized absorbing energy possessed in the atmosphere in an average climate state, was −0.23, 0.29 and 0.08 MJ m^−2^ °C^−1^ for the Dome C (2008–2011), Sodankylä (2001–2018) and QYZ (2013–2016) sites, respectively, indicating that the atmosphere at Dome C and Sodankylä have much larger stored and emitting heat capacities per unit of S/G and T than QYZ (by. about a factor of 3); Sodankylä has a little larger heat capacity than Dome C. It is probably the most important factor causing larger changes in air temperature at the two poles than at a mid-latitude site. These large differences were caused by large differences in atmospheric GLPs (e.g., chemical composition, concentration) at the three sites [17,27]. The much cleaner and drier atmosphere at the two poles makes them the most sensitive regions in terms of climate change. There is a good consistency between the above normalized absorbing energy and the annual T increase for the two poles, −0.23 and 0.29 MJ m^−2^ °C^−1^ versus 1.80 °C and 2.09 °C for Dome C (2006–2016) and Sodankylä (2001–2018), respectively, revealing the important role of the atmosphere’s stored heat capacity

The relationship between G_LA_/(S/G) and T was obtained (R = 0.48, α = 0.05, n = 19): G_LA_/(S/G) = −0.856T−20.077 (4)

**Figure 20:**
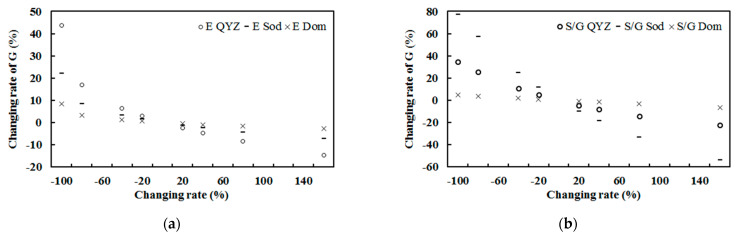
Change rates of global solar irradiance (%) due to changes in one factor (%), while other factors remain at their original levels for Dome C (expressed as Dom), Sodankylä (Sod) and QYZ. (**a**) E factor and (**b**) S/G factor.

The absorbing energy (G_LA_) for unit atmospheric GLPs is partially converted to thermal energy heating the atmosphere, and the other part is consumed in CPRs without relation to T and is constant. Similar relationships between G_LA_/(S/G) and T as in Equation (4) are acquired for Sodankylä and QYZ; the coefficients of T are 0.273 and 0.087, respectively [17,27]. The increase in absorbing solar energy of 0.856 MJ m^−2^ per unit of atmospheric GLPs can increase T by 1 °C at Dome C, and 0.273 and 0.087 MJ m^−2^ at Sodankylä and QYZ, respectively. The atmospheres at the three sites have large different normalized heat capacities, with the highest at Dome C and the smallest at QYZ. The calculated absorbing energy, thermal energy and photochemical energy increased with the increase in S/G (Figure 20).

The absorbing and scattering energy stored and utilized by GLPs, as well as the reflections (or albedos) at the TOAsur, should be considered in exploring mechanisms of climate change and for mitigating global warming.

Absorbing and scattering GLPs interact with different solar radiation components and influence energy balance at the TOAsur and in the atmosphere, as well as the climate (e.g., T, v). The GLP concentrations and their changes influence solar radiation distributions (R_LA_, R_LS_). Anthropogenic and natural activities (e.g., VOC and GLP emissions) cause additional changes in the atmosphere, biosphere and environment. To investigate the changes in T and other parameters, it is beneficial to study hourly datasets of physical, chemical and biological processes and their interactions. The GLP exchanges between atmosphere–ocean (e.g., at Dome C), atmosphere–biosphere–anthroposphere and multiple GLP—G interactions should be studied as a unified system. More and long-term measurements and model studies are necessary, including all components of solar radiation (UV, VIS, NIR, longwave radiation, etc.), and chemical compositions and meteorological variables in representative regions. Only upon comprehensive study will the unresolved mechanisms in climate change be discovered.

The absorbing GLPs attenuate more G than the scattering ones at the three sites (R_LA_ > R_LS_, Table 11). This is most evident at Dome C (R_LA_ = 95.51%, Table 11), indicating that the absorbing GLPs are dominant and play a critical role in radiative transfer and its distribution. However, this is not the case in the UV and VIS regions in north China; the annual contributions (R_LA_ and R_LS_) were 35.3% and 67.7% in the UV region and 4.7% and 95.3% in the VIS region during 2004–2006, using a similar model as Equation (1) applied in the UV and VIS regions [56,62]. More investigations are needed to understand energy distributions in all wavelength regions and the complicated laws in the sun–atmosphere–earth system.

Air masses exchanging across different regions, e.g., land–ocean, inside–outside polar regions, by transportation and atmospheric circulation are objectively considered as absorbing and scattering factors/terms, respectively. These two factors are measured and applied in hourly radiation estimations; this helps obtain reasonable albedo estimations at the TOAsur.

Sensitivity studies were also performed for Sodankylä and QYZ as for Dome C (Figure 20), using a similar model as Equation (1) and its site observations [17,27]. At the three sites, G was more sensitive to changes in S/G than in E. This feature was more prominent at Sodankylä than at Dome C and QYZ, and the ratio of the response rate to S/G to the response rate to E was 7.10 at Sodankylä, 1.63 at Dome C and 1.57 at QYZ, indicating that the atmosphere was much cleaner and drier at the two polar regions than the mid-latitude region, as S/G and E were 0.50 and 8.55 at Sodankylä, 0.135 and 0.188 at Dome C, and 0.71 and 23.96 at QYZ. (S/G)/E was 0.72 at Dome C, 0.06 at Sodankylä and 0.03 at QYZ, revealing that though there were relatively more scattering GLPs over absorbing GLPs at Dome C than Sodankylä and QYZ ((S/G)/E = 0.72, 0.06, 0.03, respectively), the empirical model can determine the relatively lower contribution of scattering GLPs to G (annual mean R_LS_ = 4.51% at Dome C, 36.68% at Sodankylä and 23.23% at QYZ for S/G < 0.80) compared to absorbing GLPs using the energy balance method (Equation (1)); this helps us accurately understand the larger contributions and more important roles of absorbing GLPs in the Antarctic region than the Arctic and mid-latitude regions. This characteristic was most obvious at Dome C, having the cleanest and driest atmosphere. It is deduced that absorbing GLPs play a more important role in the transfer and utilization of global solar radiation than scattering GLPs in most regions on the earth. The absorbing and scattering substances and their changes in concentration and composition drive the changes and distributions of G at the surface and in the atmosphere; for example, the R_LA_ values were 94.49%, 63.32% and 76.77% for Dome C, Sodankylä and QYZ, respectively, which do not correspond well to the absorbing GLP amounts (represented by E = 0.19, 8.55 and 23.96 hPa, respectively). Similar characteristics were also found in R_LS_. In addition, the lowest S/G resulted in the lowest R_LS_ at Dome C among the three regions. Compositions and their changes in GLP phases are necessary to be studied in these unique regions [27], considering that GLPs (emitted directly and produced thorough CPRs) absorb UV, VIS and NIR radiation and contribute to the climate and climate change in various ways. For example, these GLPs are commonly constituated as O_3_, NO_x_, SO_2_, CO_2_, CH_4_, N_2_O, BC, H_2_O, organic compounds, together with glyoxal, CH_3_CO radical, NO_3_ radical, OClO, CHOCHO, biacetyl, butenedial, NOCl, and thousands of VOCs [61,62,63,64,65]. Through atmospheric circulation, air-sea exchange/interaction and other processes, all GLPs (including water vapor, an important greenhouse gas) from other sites can transport into two polar regions [57,58,59,66,67]. During transportation and after arrival, multiple GLP–solar radiation interactions happen in the horizontal and vertical dimensions, from regional to global scales; so, high attention should be paid to this interaction.

The increases in absorbing and scattering GLPs at the three regions resulted in different changes in solar radiation (at the TOAsur, in the atmosphere), as well as in T, which are region- and GLP-dependent. Any larger changes in GLPs (direct emissions and secondary production) would cause large changes in solar radiation and its associated energy, and different movements of air (e.g., high/low T, v). The more GLPs and their corresponding energy remain and accumulate in the atmosphere, the more energy would be absorbed/released by GLPs with their transportation from one region to another, from the near surface to the upper atmosphere. If the previous equilibrium state between GLP–energy interactions is unable to be kept and is broken, abnormal weather or disaster may happen. It is suggested to reduce anthropogenic emissions of all sorts of GLPs (including greenhouse gases (GHGs) and non-GHGs), to help the atmosphere–anthroposphere–land system move back to its accustomed equilibrium state that can be adjusted automatically by the system itself. It will effectively contribute to UN Sustainable Development Goal 13, “taking urgent action to combat climate change”.

The responses of G to the absorbing and scattering factors between Dome C, Sodankylä and QYZ are compared in Figure 20. G_cal_ was most sensitive to changes in the absorbing factor (represented by E) at QYZ, followed by Sodankylä and then Dome C, which is caused by and well in line with the most absorbing GLPs (E) being at QYZ, then Sodankylä and finally Dome C; however, G was most sensitive to changes in the scattering factor at Sodankylä, then QYZ and finally Dome C, which corresponds to the largest total of scattering GLPs, i.e., moderate S/G multiplied by its long optical length, being at Sodankylä. Hourly mean air mass (m) multiplied by S/G values were calculated and found to be 0.86, 0.97 and 0.27 for Sodankylä (n = 3962), QYZ (n = 14, monthly data) and Dome C (n = 2771), respectively, and 3.61, 1.46 and 0.43 using hourly maximum values. Multiple scattering processes of GLPs play important roles, and accurate GLP amounts and light paths should be considered together for scattering in the real atmosphere, especially at the two poles. The least amount of scattering substances, as well as the lowest m*(S/G), at Dome C resulted in it having the minimum number of scattering losses. Similarly, hourly mean air mass multiplied by E was 32.65, 14.84 and 0.37 for QYZ, Sodankylä and Dome C, respectively. The absorbing and scattering GLP amounts, together with the real path length that photons travel, should be considered in the total absorbing and scattering processes.

### 4.6. Biogenic Secondary Organic Aerosols and Their Potential Roles

Biomass burning is a significant source of GLP emissions/formations in the atmosphere, including CO_2_, CO, NOx and BVOCs, contributing to the formation of BC and OC and impacting atmospheric chemistry and climate change [68,69,70]. BC emissions from wildfires, agricultural burning and other fires in South America, Africa and Australia can get transported to Antarctica [67].

Biomass burning enhances BVOC emissions and O_3_ concentration in forests; thus, more biogenic SOAs are produced by BVOC oxidation through OH radicals [71,72,73,74,75,76,77,78]. It contributes to the presence of cloud condensation nuclei and cloud formation, and impacts solar radiative transfer and energy balance in the atmosphere and at the ground, as well as the climate.

BVOCs, as highly reactive compounds, play significant roles in CPRs with water vapor, O_3_, NOx and all other GLPs using UV and VIS radiation. BVOCs are important reactants and connections in the changes and conversions in gas–liquid–solid substances (SOA, BC, OC, O_3_, etc.) and GLP–solar radiation interactions. Considering the absorption of major GHGs, BC and OC are not comprehensive [62,79,80,81], and it is suggested to consider BVOCs in GLP changes and energy use in BVOC–aerosol–cloud–radiation interactions.

To reduce global warming, China and other countries will attempt to achieve a goal of reaching carbon peaking and carbon neutrality in the future. One important measure is to plant large quantities of trees and grasses. More and more BVOCs will be emitted and SOA, O_3_ and other GLPs will be produced. During the COVID-19 lockdown, the UK’s surface NO_2_ dropped by 42%, but O_3_ and isoprene increased [82], revealing BVOCs’ significant roles in O_3_ formation under the new mode of anthropogenic activity. It is urgent to explore potential effects in the GLP–radiation–climate relationship caused by changes in direct emissions and the secondary production of GLPs under future anthropogenic activities.

### 4.7. Further Evaluation of the Empirical Model of Global Solar Irradiance

The sum of all coefficients and constants is the total solar irradiation at the TOA (A_1_ + A_2_ + |A_0_|), which approximately equals the solar constant I_0_ with reasonable accuracy at Sodankylä and QYZ [17,27]. For example, the ratio of A_1_ + A_2_ + |A_0_| to the solar constant is 0.999 when S/G ≤ 0.30 (sample points n = 1322) at Sodankylä. However, this ratio was 1.515 (n = 2771) using hourly data in the empirical model. This quite large ratio was mainly caused by strong reflections from the snow surface at Dome C. Considering that the net global solar irradiance at the TOA is provided from the sun, the ratio of A_1_ + A_2_ + A_0_ to the solar constant was used to evaluate the estimation of G at the TOA, and was found to be 1.069 (n = 2771), showing that the empirical model has better performance at the TOA, and the high accuracy of the solar radiation sensors/measurements over the four years. Furthermore, the EMGSI can be also used to calibrate the solar radiation sensors [83].

## 5. Conclusions

Solar energy (global, absorption, scattering, reflection, losses in the atmosphere, etc.) and all kinds of atmospheric constituents (absorbing, scattering), as well as their long-term changes, were analyzed to investigate the physical and chemical processes in the atmosphere, the climate and climate change at Dome C, Antarctica. An empirical model of global solar irradiance was developed, and good estimations of hourly global solar irradiance under all-sky conditions were manifested. Global solar irradiance at the ground and its loss in the atmosphere from 2006 to 2016 were calculated, and showed evident monthly, annual and interannual variations. A sensitivity test showed that global solar irradiance is more sensitive to changes in scattering than absorption, with nonlinear and negative responses of global solar irradiance to changes in the absorbing and scattering factors. The estimated albedos at the TOAsur agreed with the satellite-retrieved values.

During 2006–2016, the estimated annual global solar irradiance decreased by 0.09% and the diffuse irradiance increased by 0.68% per year, associating them with increases in S/G by 0.57% and E by 1.46% per year. Annual air temperature increased by 1.80 °C. The annual mean absorbing, scattering and total losses of global solar irradiance in the atmosphere were 4.02, 0.19 and 4.21 MJ m^−2^, respectively, and increased by 0.01%, 0.39% and 0.28% per year, respectively. The contributions of the annual mean absorbing and scattering losses to the total loss were about 96% and 4%, respectively, meaning that the absorbing substances/processes have dominant roles. The estimates of TOA albedos were smaller than that of the surface albedos. The estimated and satellite-retrieved annual albedos showed a very small decrease at the TOA and a slight increase at the surface.

The global solar radiation and its components, the air temperature and other key factors at Dome C, Sodankylä and QYZ were analyzed. Global solar radiation received in the atmosphere and its interactions with GLPs play different but controlling roles in regional climates and climate change, as well as in air temperature changes.

## Figures and Tables

**Table 1:** Main technical parameters/protocols of measurements at Dome C.

Parameter	Instrument	Protocols	Surroundings
G	CM22, Kipp & Zonen Inc.	BSRN	homogeneous snow surface
S	CM22, Kipp & Zonen Inc.	BSRN	temperature: −80 °C to −20 °C
D	CH1, Kipp & Zonen Inc.	BSRN	cold, dry, clear and clean atmosphere
T, RH, v	automatic weather station	http://www.climantartide.it (accessed on 17 January 2022)	very low AOD

**Table 2:** The coefficients and constants (MJ m^−2^), coefficient of determination (R^2^), average and maximum of the absolute relative bias (δ_avg_, δ_max_ (%)), NMSE (MJ m^−2^) and standard deviations of calculated and observed solar global exposures (σ_cal_ and σ_obs_, respectively, MJ m^−2^). The mean bias errors (MAD, MJ m^−2^ and %) and the root mean square errors (RMSE, MJ m^−2^ and %) (n = 2771).

A_1_	A_2_	A_0_	R^2^	δ_avg_	δ_max_	NMSE	σ_cal_	σ_obs_	MAD		RMSE	
									(MJ m^−2^)	(%)	(MJ m^−2^)	(%)
5.607	0.752	−1.097	0.993	1.76	5.23	0.0004	0.500	0.502	0.036	1.68	0.043	2.02

**Table 3:** The observed and calculated monthly global solar exposure (MJ m^−2^), average and maximum of the absolute relative bias (δ_avg_, δ_max_ (%)), NMSE (MJ m^−2^) and standard deviations of calculated and observed hourly mean solar global exposures (σ_cal_ and σ_obs_, respectively, MJ m^−2^). The mean bias errors (MAD, MJ m^−2^ and %) and the root mean square errors (RMSE, MJ m^−2^ and %). All values are for monthly averages. The solar altitude angle is h (degrees) (n = 62).

h	G_obs_	G_cal_	δ_avg_	δ_max_	NMSE	σ_cal_	σ_obs_	MAD		RMSE	
								(MJ m^−2^)	(%)	(MJ m^−2^)	(%)
≥5°	1.333	1.270	4.86	11.07	0.00003	0.836	0.750	0.064	4.81	0.007	0.56
≥10°	1.555	1.533	2.15	6.05	0.00001	0.280	0.296	0.034	2.21	0.004	0.23

**Table 4:** Change rate (%) of the monthly mean solar radiation and meteorological variables (air temperature T and its change ΔT (°C), relative humidity RH (%), water vapor pressure E (hPa)) during January–March and October–December period across 2006–2016. h is the solar altitude (degrees). Change rate of each variable was calculated using c_1_ × 100/c_0_, and linear relation between each variable (y) and time (month, x) was determined as y = c_1_x + c_0_.

h	G_obs_	G_cal_	S	T	ΔT (°C)	RH	E	S/G	G_LA_	G_LS_	G_L_
≥5°	0.002	0.006	0.132	0.04	0.78	0.19	0.48	0.07	−0.004	0.039	−0.002
≥10°	0.013	0.003	0.135	0.05	1.07	0.18	0.54	0.04	−0.003	0.040	−0.001

**Table 5:** Correlations among monthly solar exposure (observed and calculated, G_obs_ and G_cal_, losses due to absorbing and scattering and total loss, G_LA_, G_LS_, G_L_) and meteorological variables (air temperature T, relative humidity RH, water vapor pressure E, air pressure P, wind speed v, S/G) during January–March and October–December in 2006–2016.

	T-G_obs_	T-G_cal_	T-G_LA_	T-G_LS_	T-G_L_	T-S/G	G_LA_-E	G_LS_-E	G_L_-E	G_LA_-S/G	G_LS_-S/G	G_L_-S/G	P-S/G	v-S/G	P-E
≥5°	0.844	0.797	−0.829	0.042	−0.797	0.145	0.717	0.147	0.676	0.147	0.985	0.256	0.098	0.020	0.730
≥10°	0.864	0.826	−0.852	0.050	−0.826	0.155	0.739	0.162	0.704	0.114	0.986	0.210	0.040	0.044	0.726

**Table 6:** Monthly and annual averages (MAVG, AAVG) of solar radiation and meteorological parameters calculated from hourly values for h ≥ 5° and h ≥ 10° during January–March and October–December in 2006–2016. h is the solar altitude (degrees).

Average	h Degree	G_obs_ MJ m^−2^	G_cal_ MJ m^−2^	S MJ m^−2^	T °C	RH %	E hPa	S/G	G_LA_ MJ m^−2^	G_LS_ MJ m^−2^	G_L_ MJ m^−2^	R_LA_ %	R_LS_ %
MAVG	≥5°	1.333	1.270	0.332	−41.84	56.86	0.120	0.296	3.889	0.149	4.038	95.58	4.42
MAVG	≥10°	1.555	1.533	0.373	−41.06	57.32	0.129	0.268	3.564	0.164	3.729	95.64	4.39
AAVG	≥5°	1.328	1.265	0.332	−41.77	56.93	0.120	0.297	3.818	0.180	3.997	95.56	4.44
AAVG	≥10°	1.549	1.528	0.374	−40.99	57.40	0.129	0.270	3.569	0.165	3.374	95.62	4.38

**Table 7:** Change rate of G (%) due to changes in E or S/G (%), with S/G and E retaining their original values. Change rate of G was calculated using (G_caln_ − G_cal_) × 100/G_cal_, G_caln_ was G_cal_ using new E or S/G and G_cal_ was the previous estimation using original values of E and S/G.

E (%)							S/G (%)							
+20	+40	+80	+160	−20	−40	−80	+20	+40	+80	+160	−20	−40	−80	−100
−0.48	−0.91	−1.66	−1.85	0.56	1.22	3.28	−0.89	−1.76	−3.42	−6.48	0.92	1.86	3.84	4.87

**Table 8:** Change rates (%) of observed and calculated G (G_obs_, G_cal_), absorbing, scattering and total losses (G_LA_,G_LS_, G_L_) of global solar exposure due to GLPs, air temperature (T), water vapor pressure (E), scattering factor (S/G) over different time periods in 2006–2016.

G_obs_	G_cal_	G_LA_	G_LS_	G_L_	T	E	S/G	n
1.4 × 10^−3^	1.4 × 10^−3^	−1.0 × 10^−4^	−1.1 × 10^−3^	−1.0 × 10^−3^	2.9 × 10^−3^	6.0 × 10^−3^	1.4 × 10^−3^	2771
5.4 × 10^−4^	3.8 × 10^−4^	−3.4 × 10^−4^	3.0 × 10^−3^	−2.0 × 10^−4^	2.8 × 10^−3^	3.0 × 10^−5^	3.9 × 10^−3^	6356
1.5 × 10^−5^	1.6 × 10^−6^	−5.2 × 10^−6^	5.6 × 10^−5^	−5.0 × 10^−7^	1.6 × 10^−4^	5.7 × 10^−4^	9.9 × 10^−5^	33311

**Table 9:** As in Table 8, but for annual average change rate (%) calculated using monthly average in JFND during 2006–2016.

G_obs_	G_cal_	G_LA_	G_LS_	G_L_	T (% and °C)	E	S/G	n
−0.06	−0.19	0.03	1.50	0.09	0.58 (2.12 °C)	3.56	2.10	2771

**Table 10:** Averages of observed G (G_obs_), T, E, and S/G for 3 situations.

G_obs_ (MJ m^−2^)	T (°C)	E (hPa)	S/G	n	Situation
2.14	−35.94	0.188	0.135	2771	1
1.87	−37.32	0.184	0.261	6356	2
1.34	−41.55	0.135	0.308	33311	3

**Table 11:** Annual averages of observed monthly meteorological variables and S/G, simulated monthly global solar radiation and its loss, albedos at the TOA and the surface at Sodankylä (refer to as Sod), Dome C (Dome) and QYZ sites under all skies during 2013–2016, and the ratios of all parameters between Sodankylä and QYZ and between Dome C and QYZ (ratio 1 and ratio 2) (alb and sur denote albedo and surface, respectively).

Site	G_cal_ MJ m^−2^	T °C	RH %	E hPa	S/G	G_LA_ MJ m^−2^	G_LS_ MJ m^−2^	G_L_ MJ m^−2^	G_LA_ Wm^−2^	G_LS_ Wm^−2^	G_L_ Wm^−2^	R_LA_ %	R_LS_ %	alb TOA	alb sur
Sod	0.65	3.05	76.00	6.83	0.59	1.94	1.23	3.18	539.65	342.54	882.19	61.96	38.04	0.36	0.22
QYZ	1.42	22.71	75.76	22.38	0.83	1.68	0.27	1.95	466.37	75.35	541.71	89.31	13.69	0.29	0.22
Dome	1.25	−41.39	58.23	0.13	0.31	3.83	0.18	4.01	1064.06	50.61	1114.85	95.51	4.49	0.69	0.80
Ratio1	0.46	0.13	1.00	0.30	0.71	1.15	4.56	1.63	1.16	4.55	1.63	0.69	2.77	1.24	0.99
Ratio2	0.88	−1.82	0.37	0.006	0.37	2.28	0.67	2.06	2.28	0.67	2.06	1.07	0.33	2.38	3.64

**Table 12:** Change rate (%) of annual monthly averages of solar radiation and meteorological variables, and calculated albedos at the TOA and the surface at Dome C, Sodankylä and QYZ sites under all skies during 2013–2016.

Site	G_cal_	G_obs_	T	RH	E	S/G	G_LA_	G_LS_	G_L_	alb TOA	alb sur
Sod	−0.54	−2.45	−3.79	0.17	1.13	0.86	0.03	0.25	0.11	−1.20	−0.11
QYZ	−3.93	−5.55	−1.96	4.34	0.50	6.08	0.01	−9.71	−1.72	11.73	−0.81
Dome	−1.25	−0.85	−1.25	0.87	−5.88	3.34	0.15	3.13	0.27	0.77	2.10

## Data Availability

Not applicable, the Dome C data in minute resolution can be found at https://doi.org/10.1594/PANGAEA.935421, accessed on 17 January 2022.
